# Production of Platelet-Rich Gel Using Allogeneic Platelets Isolated from Donor Whole Blood

**DOI:** 10.17691/stm2025.17.6.04

**Published:** 2025-12-29

**Authors:** M.S. Makarov, M.V. Storozheva

**Affiliations:** DSc, Senior Researcher, Scientific Department of Biotechnologies and Transfusiology; N.V. Sklifosovsky Research Institute for Emergency Medicine, Healthcare Department of Moscow, 3 Bolshaya Sukharevskaya Sq., Moscow, 129090, Russia; Researcher, Scientific Department of Biotechnologies and Transfusiology; N.V. Sklifosovsky Research Institute for Emergency Medicine, Healthcare Department of Moscow, 3 Bolshaya Sukharevskaya Sq., Moscow, 129090, Russia

**Keywords:** allogeneic platelets, centrifugation, platelet gel, morphofunctional status of platelets

## Abstract

**The aim of the study** was to perform a morphofunctional analysis of allogeneic platelets isolated from donor whole blood and to assess the possibility of producing platelet-rich gel based on them.

**Materials and Methods:**

The study investigated donor blood platelets, platelets collected from donors via apheresis, and platelets isolated from donor whole blood (platelet-leukocyte concentrates, PLC). Platelet quality was assessed before and after centrifugation at 2500–4000 g, as well as the feasibility of producing platelet gel from platelet-rich plasma isolated from whole blood. Morphofunctional analysis of platelets was performed using an original method based on the examination of vitally stained cells by fluorescence microscopy. The cytokine profile in the platelet concentrates isolated from whole blood was evaluated using multiplex analysis.

**Results:**

Following centrifugation at 2500–4000 g, the platelet population exhibited an increased number of platelets with damaged membranes, procoagulant platelets, and platelets prone to spontaneous activation. Centrifugation at 2500–2700 g was less damaging than centrifugation at 2701–4000 g and allowed to preserve a significant volume of growth factors within the platelets. Platelet-rich plasma isolated from PLC can be used to produce platelet gel and thrombofibrin clot *in vitro* at 20–22°C without the use of platelet activation inducers. The most effective PLC samples for producing platelet gel were those without platelet conglomerates, containing over 20% platelets with granules and less than 20% platelets with damaged membranes, with a level of procoagulant platelets below 5%, and with an increased or high adhesion rate of platelets with granules to glass. The use of platelets from allogeneic PLC enables the production of platelet gel within 20–30 min.

**Conclusion:**

There was developed a method for producing platelet gel and a thrombofibrin clot at 20–22°C based on allogeneic platelets isolated from donor whole blood. These materials can be used as wound dressings, applicative biological constructs for treating tissue defects of various origins, and in the creation of composite biological designs intended for use in regenerative medicine.

## Introduction

Human platelets have a unique and diverse biological potential that can be used for various tasks in regenerative medicine [1, 2]. Platelet gel (PG) is one of the widely used biological preparations. PG is formed as a result of platelet activation *in vitro* and has a gel-like structure containing growth factors and other biologically active components secreted by platelets [3]. PG is a convenient material for creating wound covers and applicative bioconstructions and can be effective in the production of composite cell-tissue constructs and other biotechnological products [4]. Generally, autologous plasma isolated from the patient’s blood is used to prepare PG. However, in some cases, a patient’s own platelets have a reduced or low biological potential, as well as structural and functional impairments [5], which may make PG based on them ineffective. Preparations based on allogeneic platelets are currently rarely used for tissue regeneration; nevertheless, allogeneic platelets and their concentrates are of interest to regenerative medicine. In particular, one potential source of platelet components is platelet-leukocyte concentrates (PLC) isolated during the separation of donor whole blood. PLC are used to obtain pooled platelet concentrates for use in clinical transfusiology [6]. Moreover, this component can serve as a starting material for creating platelet preparations [7]. Allogeneic platelets isolated from donor whole blood represent a valuable reservoir of growth, reparative, and regenerative factors and can be used, among other purposes, for creating applicative biological products.

Nowadays, there are no standardized methods for obtaining applicative biological products, specifically PG and thrombofibrin clot, and there is no precise understanding of the quality of allogeneic platelets used for creating these preparations [8]. PG is often prepared by preliminary freezing of platelet-rich plasma followed by plasma activation with calcium preparations at temperature of 37°C [9–11]. However, this approach is accompanied by the loss of many growth factors and does not allow the usage of the functional features of platelets during PG formation due to their cryodestruction. Therefore, the search for methods aimed to improving the efficiency of selecting and using allogeneic platelets in the creation of applicative biological preparations is up-to-date.

**The aim of the study** was to determine the morphofunctional characteristics of allogeneic platelets isolated from donor whole blood and to assess the possibility of producing platelet gel based on them.

## Materials and Methods

We studied blood components from 10 donors and 10 volunteer donors, collected into 4–5 ml tubes with the anticoagulants citrate or ethylenediaminetetraacetic acid (EDTA). Platelets prepared via automated apheresis platelet concentrates (APC; 20 donors) with citrate-phosphate-dextrose (CPD) or sodium saline phosphate (SSP) preservative solutions, and platelets isolated as part of PLC from whole blood (20 donors) were also studied. The research was conducted with the approval of the Ethics Committee of the N.V. Sklifosovsky Research Institute for Emergency Medicine (local ethics committee protocol No.8-23, dated October 11, 2023) and in accordance with the Helsinki Declaration (2024). Informed consent for research participation was obtained from all the donors.

The Haemonetics MCS+ (Haemonetics Corporation, USA) or Trima Accel (Terumo BCT, USA) systems were used to obtain APC; the Reveos system (Terumo BCT, USA) was used to obtain PLC. Donor and volunteer whole blood platelets and apheresis-derived platelets were analyzed immediately after blood collection and after 2 days of storage on a shaker at 20–22°C. To assess the impact of “harsh” centrifugation on platelet quality *in vitro*, blood samples were centrifuged at 2500–4000 g for 10 min, after which the platelet-leukocyte layer, analogous to PLC, was collected. A 10% calcium chloride solution and a 1 mg/ml adrenaline solution were used to produce PG from PLC *in vitro*. For platelet activation, 5–7 μl of calcium chloride and 1 mM adrenaline were added per 95–100 μl of PLC.

Morphofunctional analysis of platelets was performed using an original method based on the examination of vitally stained cells with fluorescence microscopy [12]. For this purpose, cells were stained with a vital (lifetime) fluorochrome dye based on trypaflavine and acridine orange. A Nikon Eclipse 80i confocal microscope coupled with a Nikon Intensilight C-HGFI fluorescence lamp (Nikon Corporation, Japan) was used.

The following parameters were analyzed to assess cell quality in PLC: platelet concentration; leukocyte concentration; the level of platelets with granules (GPL), normal range 35–75%; the proportion of adhesively active platelets (AAP), normal range 30–75%; the level of platelets with damaged membranes (PDM), normal range 2–3%; the level of procoagulant platelets (COAGT), which are normally absent in the blood of healthy individuals. PDM do not have granules and are functionally inactive. COAGT represent a distinct morphological platelet type formed at the terminal stage of platelet activation. COAGT exhibit massive deformation of intracellular structures with displacement to the periphery, forming a characteristic morphological structure resembling a protrusion (“cap”) or a narrow cytoplasmic crescent. The main volume of such platelets has very low optical density [13]. We also assessed the presence of platelet conglomerates in the sample; the pattern of granule distribution within platelets was studied; the presence of platelets with altered shape indicating their activation was noted; and the rate of platelet adhesion to glass. Normally, the duration of adhesion for a single platelet is 10–30 min from the moment the platelet suspension is applied to the glass. During this time, a change in platelet shape and the release of platelet granules occur. If these processes occur within 5 to 10 min, the adhesion rate is considered increased; 3 to 5 min — high; up to 3 min — very high; and more than 30 min — low. The cytokine profile was assessed in the initial PLC samples, as well as in the platelet-poor plasma obtained after sedimentation of PLC platelets. To obtain platelet-poor plasma, the initial PLC samples were centrifuged at 700 g for 10–17 min. The cytokine profile was studied using multiplex analysis on the Luminex 200 platform (xMAP technology) with the MILLIPLEX MAP Human Cytokine/Chemokine Magnetic Bead Panel kit (Merck Millipore, Germany). The concentrations of platelet-derived growth factor (PDGF AB/BB), fibroblast growth factor (FGF-2), epidermal growth factor (EGF), interleukins 1-beta (IL-1β) and 8 (IL-8), and vascular endothelial growth factor (VEGF) were determined.

***Statistical analysis*** of the data was made using Statistica 10.0 and BioStat Pro 5.9.8 software. Normality of distribution was tested using the Kolmogorov–Smirnov test. Given the high variability of platelet morphofunctional parameters, nonparametric statistical criteria were used. For statistical processing, the median (Me) and the 1^st^ and 3^rd^ quartiles [25%; 75%] were calculated. The Mann–Whitney U test for independent variables and the Wilcoxon test for related samples were used to compare quantitative data between two independent groups. The level of statistical significance (p) and the adjusted level of statistical significance using the Bonferroni correction (p_adjusted_) for multiple comparisons were determined. Differences were considered statistically significant at p<0.05.

## Results

**Figure 1.**
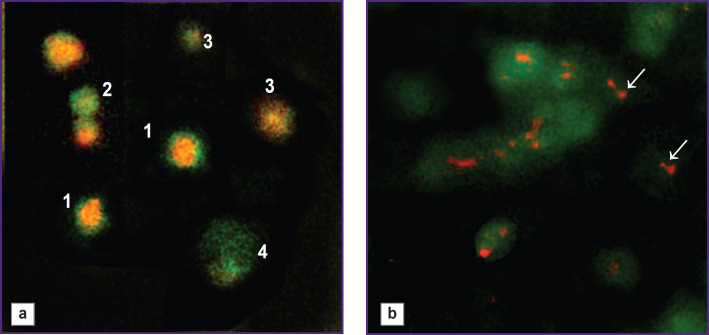
Impact of centrifugation at 2500–2700 g on the morphofunctional characteristics of platelets: (a) different morphological types of platelets: *1* — platelet with granules, *2* — degranulated platelet, *3* — platelet with damaged membranes, *4* — procoagulant platelet; (b) partial extrusion of granules from platelets and free platelet granules (*arrows*). Vital staining with trypaflavine and acridine orange; ×1500

Our previous research has demonstrated that “harsh” centrifugation (3000–4000 g) of apheresis-derived platelets reduces their quality and ability to maintain functional and structural characteristics over time [14]. However, that study did not account for the platelets’ capacity to form a thrombofibrin clot or the presence of hyperreactive cells within the platelet population. The present study has established that increasing the relative centrifugal force during *in vitro* centrifugation leads to greater losses in platelet quality (Table 1). In 30–35% of cases, centrifugation at 2500–4000 g resulted in the formation of platelet conglomerates — structures formed by platelet clumping, similar to platelet aggregates. Conglomerates differ from aggregates by their lower cell density and the absence of morphological changes characteristic of irreversible platelet activation. These platelet conglomerates can be very large (up to 100 μm). In the studied samples, the conglomerates consisted of degranulated cells and did not dissociate even with prolonged mixing. The level of GPL in such samples did not exceed 15–16% in any case, often being less than 10%, indicating very low platelet quality. Massive platelet degranulation suggests their spontaneous activation during the initial blood separation stage; consequently, these platelets exhibited low biological and functional activity after isolation. Furthermore, “harsh” centrifugation led to the formation of COAGT (Figure 1 (a)), which possess the ability to adsorb plasma hemostasis components. These platelets are simultaneously irreversibly activated and apoptotic cells, they contain no granules or growth factors [15]. COAGT can participate in platelet aggregation but are also capable of transmitting pro-apoptotic signals to unactivated, normal platelets, thereby increasing the risk of damage to the entire platelet population and loss of their biological potential.

**T a b l e 1:** Effect of centrifugation regimens on platelet quality and activity

Parameters		Apheresis-derived platelets (n=20)		Platelets after “harsh” centrifugation (n=20)		
Maximum relative centrifugal force (g)		1000–1500	1000–1500	2500–2700	2701–3000	3001–4000
Anticoagulant		SSP	CPD	Citrate/EDTA	Citrate/EDTA	Citrate/EDTA
Level of platelets with granules (%), Me [25%; 75%]	Immediately after collection	50 [46; 55]	52 [48; 56]	48 [42; 52]	48 [42; 51]	44 [38; 49]
	After 2 days of storage	42 [39; 46]*	43 [40; 46]*	21 [18; 25]*	19 [16; 23]*	11 [9; 12]*#
Presence of platelet conglomerates	No (% of cases)	100	100	65	70	70
	Yes (% of cases)	0	0	35	30	30
Level of procoagulant platelets	No (% of cases)	100	100	10	10	0
	Yes (% of cases)	0	0	90	90	100
	% of total platelet population, Me [25%; 75%])	0 [0; 0]	0 [0; 0]	5 [4; 6]	6 [4; 7]	8 [4; 12]#
Formation of platelet gel at 20–22°C (activator — 10% calcium chloride)	No (% of cases)	100	100	70	80	90
	Yes (% of cases)	0	0	30	20	10

* p_adjusted_<0.0025 compared to initial samples;

# p_adjusted_<0.0025 compared to centrifugation at 2500–2700 g.

COAGT were absent in initial donor blood and APC. In samples subjected to “harsh” centrifugation, their levels varied from 3 to 10%, with an increase in the proportion of COAGT accompanied by a decrease in GPL and AAP. It is important to note that 15–20% of APC samples showed accelerated platelet adhesion upon application to a glass slide, often accompanied by the formation of small, flattened aggregates within 5–15 min of platelet contact with the glass. This could be due to either initially high donor platelet reactivity or pre-activation of platelets during the apheresis procedure. Yet all the donor blood and APC samples did not contain COAGT, with GPL and AAP levels within the normal range, and the PDM level not exceeding 2–4%. Nevertheless, even in APC samples with increased platelet adhesion rates, it was never possible to produce PG using 10% calcium chloride at 20–22°C. Thus, the potential platelet pre-activation in APC was insufficient to activate the entire population under these conditions. Notably, in some samples after “harsh” centrifugation, it was possible to achieve PG formation at 20–22°C using only calcium chloride; however, as the centrifugation speed increased, the levels of PDM and COAGT decreased. In samples with COAGT levels above 5%, PG formation at 20–22°C was possible in some cases, but under these conditions, the gel formation time was consistently longer than at 37°C using standard methods. Therefore, to obtain PG from PLC, platelets must be isolated from whole blood at 2500–2700 g. It is worth mentioning that regardless of the g-force, successful gel formation was achieved in less than 40% of the samples. This indicates the necessity of selecting PLC samples based on a detailed analysis of the morphofunctional status of their constituent platelets.

In PLC samples, the platelet and leukocyte concentrations were 1870 [1650; 2050]·10^9^/L and 6.0 [5.0; 7.5]·10^9^/L, respectively; after centrifuging PLC at 300–500 g to sediment leukocytes, the platelet concentration in the final platelet-rich plasma remained unchanged, and the leukocyte concentration did not exceed 0.1·10^9^/L. However, the morphofunctional status of PLC platelets was initially reduced compared to the norm: GPL values were 24 [17; 25]%, AAP were 22 [16; 25]%, PDM were 15 [13; 24]%, and COAGT were 7 [5; 10]%. Among the PLC, there were doses with a very low granulated platelet count (less than 10%) and doses with a platelet count close to normal (29–30%). Among the granulated platelets, many cells had an altered pattern of granule distribution within the cytoplasm — granules were displaced from the granulomere region to the cell periphery (see Figure 1 (a)), granules were bound to the plasma membrane, and there was partial “extrusion” of large granules beyond the cytoplasm without complete degranulation (Figure 1 (b)). These platelets exhibited an increased adhesion rate on the glass slide. In addition, PLC contained free platelet granules up to 600 nm in diameter. Platelet granules can stimulate blood coagulation processes independently of platelets and adhere to fibrin and collagen. The presence of a large number of free platelet granules in the plasma increases its coagulogenic potential. Intact platelet granules also contain many growth factors [16]. Thus, it can be concluded that PLC platelets potentially have increased reactivity despite their reduced morphofunctional characteristics.

Cytokine analysis showed that growth factors were present in both PLC and the platelet-poor plasma derived from it; the levels of pro-inflammatory cytokines did not differ significantly between PLC and platelet-poor plasma (Table 2), confirming the release of a portion of platelet granules beyond the platelets during PLC preparation. Nevertheless, the levels of the growth factors EGF, FGF-2, and VEGF exceeded those in platelet-poor plasma by 3.5–7.0-fold, and PDGF AB/BB by 1.7-fold (p_adjusted_=0.001). Consequently, a significant proportion of growth factors is retained within the platelets even after “harsh” centrifugation at 2500–2700 g.

**T a b l e 2:** Cytokine profile of plasma with allogeneic platelets isolated from donor whole blood at 2500–2700 g

Preparation type	Cytokine concentration (pg/ml), Ме [25%; 75%] (n=10)					
	PDGF AB/BB	EGF	FGF-2	IL-1β	IL-8	VEGF
PLC	30,214 [27,095; 36,058]	2050 [1290; 2481]	1822 [901; 3100]	12 [10; 16]	251 [135; 368]	668 [125; 1162]
Platelet-poor plasma derived from PLC	18,197 [17,128; 22,916]*	455 [262; 650]*	514 [421; 581]*	13 [10; 18]	284 [135; 382]	83 [0; 381]*

* relative to PLC at p_adjusted_<0.005. PLC — platelet-leukocyte concentrates.

Plasma, containing allogeneic platelets isolated from PLC, represents a potentially rich source of cell growth factors, as well as reparative and regenerative factors. However, any additional manipulation of the platelet-rich plasma can lead to the loss and degradation of these factors. Therefore, to obtain a growth factor-saturated PG and thrombofibrin clot from PLC allogeneic platelets, it is necessary to minimize plasma manipulation if it is possible.

It was essential to determine the possibility of gel producing at room temperature (20–22°C) from plasma containing allogeneic platelets, derived from PLC. We had previously demonstrated the possibility of generating PG at 20–22°C using adrenaline at a final concentration of 1 mM [17]. Under conditions of increased platelet reactivity, calcium chloride can affect both the plasma and cellular components of hemostasis. The potential for activating platelets isolated from PLC under the influence of calcium chloride with 1 mM adrenaline, as well as calcium chloride alone, was investigated. It was found, that in plasma with a platelet concentration of 1700·10^9^/L and 24–25% of GPL PG formed both in the presence and absence of adrenaline, with gelation times of 16 and 24 min, respectively. It was shown, that plasma with allogeneic platelets formed a PG at a specific ratio of plasma to 10% calcium chloride. To obtain the PG it is necessary to mix 5–7 μl of 10% calcium chloride with 95–100 μl of plasma containing 24–25% of GPL. Under these conditions, the gel formed within 20–30 min. At other ratios, the gel formed much more slowly or did not form at all. Thus, the ratio of 5–7 μl of 10% calcium chloride to 95–100 μl of platelet-rich plasma is optimal.

**Figure 2.**
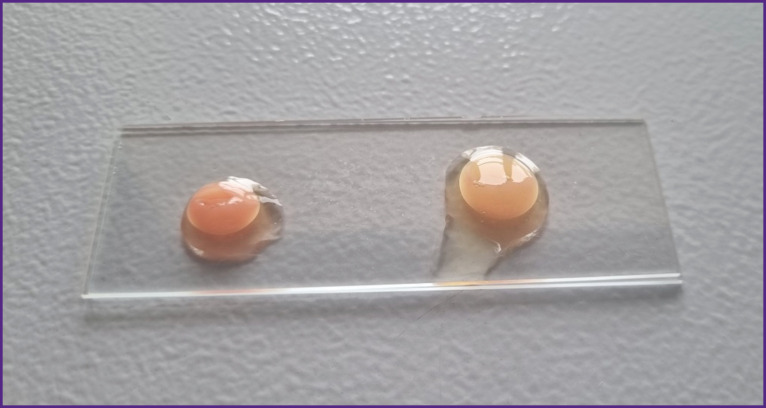
Samples of thrombofibrin clot obtained from plasma with allogeneic platelets isolated from donor blood thromboleukocyte concentrates: left — clot derived from plasma containing 21% platelet with granules, 14% platelets with damaged membranes, 4% procoagulant platelets, with an increased platelet adhesion rate; right — clot derived from plasma containing 25% platelet with granules, 9% platelets with damaged membranes, 3% procoagulant platelets, with an increased platelet adhesion rate. Platelet concentration in the plasma was (1800–1900)·10^9^/L

The study of samples isolated from different PLC units demonstrated, that the gelation rate varied considerably depending on platelet quality. In 80% of cases, samples with a GPL level lower 20% failed to form a gel; furthermore, in 20% of cases, PG formation required a very prolonged incubation (over 60 min). The presence of platelet conglomerates, a large number of platelets with severe membrane defects (20% or more of the total platelet population), and COAGT (5% or more) in the plasma also reduced the PG formation rate and could completely prevent gelation. The most effective gel formation occurred in samples devoid of platelet conglomerates, containing more than 20% platelets with granules, PDM level below 20%, COAGT level below 5%, and with an increased or high adhesion rate of platelets with granules to glass — under these conditions, the gelation time at 20–22°C was 20–30 min (Figure 2) and was comparable to that observed when producing a PG from plasma with normal platelet quality [17]. Within 10–20 min after gel formation, the process of retraction (shrinking) begins, leading to the formation of a thrombofibrin clot. Under strong mechanical stress, the thrombofibrin clot forms almost instantly. The thrombofibrin clot maintains a gel-like consistency and contains the main volume of growth factors secreted by the platelets.

## Discussion

Gel-like structures based on platelet-rich plasma formed *in vitro* are promising for application in regenerative medicine due to the biological and reparative potential of platelets. PG can be obtained, among other methods, from non-preserved blood via centrifugation [18]. However, a characteristic feature of this gel is its rapid retraction with serum formation, into which a significant portion of platelet components is released, while the level of growth factors in the serum is markedly reduced compared to the initial platelet suspension [2, 16]. Incorporating platelets or their components within various matrices enhances the stability and efficacy of growth factors and other biologically active substances secreted by platelets [19–20].

Our previous studies demonstrated that platelet activation at 20–22°C was accompanied by slower degranulation and promoted the fixation of exocytosed platelet granules on collagen or fibrin [5, 16]. Producing a PG at 20–22°C increases the saturation of the final thrombofibrin clot with growth factors. Furthermore, in a number of cases, the use of standard inducers that interact with plasma membrane receptors is not required for platelet activation. Platelets are highly sensitive and reactive cells capable of rapid responses to changes in the physicochemical conditions of their environment [21–23]. The current study has established that centrifuging platelets at 2500–2700 g reduces the proportion of biologically competent cells and at the same time can induce their pre-activation, i.e., a transition into a state of increased reactivity and sensitivity. Centrifugation induces partial platelet degranulation, which increases the plasma concentration of secreted procoagulant factors and promotes platelet activation at 20–22°C. Additionally, a portion of platelet granules is released as intact microparticles, which can also enhance platelet activity. This makes it possible to obtain a PG at 20–22°C without using inducers that directly activate platelets.

## Conclusion

PLC isolated from whole blood by centrifugation at 2500–2700 g exhibit a reduced content of biologically competent platelets, which in some cases is accompanied by their increased reactivity. This high platelet reactivity enables the production of platelet gel from PLC at 20–22°C. The resulting platelet gel and thrombofibrin clot, produced based on allogeneic platelets from donor blood PLC, can be used as wound dressings, applicative biological designs for the treatment of tissue defects of various origins, as well as in the development of composite biological constructs intended for use in regenerative medicine.

## References

[1] Golebiewska E.M., Poole AW (2013). Secrets of platelet exocytosis — what do we really know about platelet secretion mechanisms?. Br J Haematol.

[2] Amable P.R., Carias R.B., Teixeira M.V., da Cruz Pacheco I., Corrêa do Amaral R.J., Granjeiro J.M., Borojevic R (2013). Platelet-rich plasma preparation for regenerative medicine: optimization and quantification of cytokines and growth factors.. Stem Cell Res Ther.

[3] Palumbo V.D., Rizzuto S., Damiano G., Fazzotta S., Gottardo A., Mazzola G., Lo Monte A.I (2021). Use of platelet concentrate gel in second-intention wound healing: a case report.. J Med Case Rep.

[4] Everts P.A., Lana J.F., Alexander R.W., Dallo I., Kon E., Ambach M.A., van Zundert A., Podesta L (2024). Profound properties of protein-rich, platelet-rich plasma matrices as novel, multi-purpose biological platforms in tissue repair, regeneration, and wound healing.. Int J Mol Sci.

[5] Makarov M.S (2018). Physiological and prognostic value of platelets without granules.. Medicinskij alfavit.

[6] Rozhkov E.V., Kozhemyako O.V., Ponasenko M.A., Karaseva I.A., Rozhkova N.S., Madzaev S.R., Zhiburt E.B (2022). Improvement of pooled pathogen-reduced platelets concentrate production.. Transfuziologiya.

[7] Akbarzadeh S., McKenzie M.B., Rahman M.M., Cleland H (2021). Allogeneic platelet-rich plasma: is it safe and effective for wound repair?. Eur Surg Res.

[8] Asadi M., Alamdari D.H., Rahimi H.R., Aliakbarian M., Jangjoo A., Abdollahi A., Bahar M.M., Azadmand A., Forghani N., Sadegh M.N., Khayamy M.E., Seifalian A (2014). Treatment of life-threatening wounds with a combination of allogenic platelet-rich plasma, fibrin glue and collagen matrix, and a literature review.. Exp Ther Med.

[9] Wang S., Ding W., Du Y., Qi Q., Luo K., Luan J., Shen Y., Chen B (2023). Allogeneic platelet gel therapy for refractory abdominal wound healing: a preliminary study.. Adv Clin Exp Med.

[10] Fujioka-Kobayashi M., Schaller B., Mourão C.F.A.B., Zhang Y., Sculean A., Miron R.J (2021). Biological characterization of an injectable platelet-rich fibrin mixture consisting of autologous albumin gel and liquid platelet-rich fibrin (Alb-PRF).. Platelets.

[11] Fan Y., Perez K., Dym H (2020). Сlinical uses of platelet-rich fibrin in oral and maxillofacial surgery.. Dent Clin North Am.

[12] Makarov M.S., Khubutia M.Sh., Khvatov V.B., Vysochin I.V., Kobzeva E.N., Konyushko O.I (2013). A method for assessing the morphofunctional status of human platelets. Patent RU 2485502..

[13] Podoplelova N.A., Sveshnikova A.N., Kotova Y.N., Eckly A., Receveur N., Nechipurenko D.Y., Obydennyi S.I., Kireev I.I., Gachet C., Ataullakhanov F.I., Mangin P.H., Panteleev M.A (2016). Coagulation factors bound to procoagulant platelets concentrate in cap structures to promote clotting.. Blood.

[14] Makarov M.S., Borovkova N.V., Khvatov V.B., Kobzeva E.N (2015). The effect of centrifugation on the biological usefulness of human platelets.. Vestnik sluzby krovi Rossii.

[15] Denorme F., Campbell R.A (2022). Procoagulant platelets: novel players in thromboinflammation.. Am J Physiol Cell Physiol.

[16] Italiano J.E., Mairuhu A.T., Flaumenhaft R (2010). Clinical relevance of microparticles from platelets and megakaryocytes.. Curr Opin Hematol.

[17] Makarov M.S., Storozheva M.V., Borovkova N.V., Ponomarev I.N (2019). A method for preparing a thrombofibrin clot with growth-stimulating properties. Patent RU 2679616..

[18] Santhakumar M., Yayathi S., Retnakumari N (2018). A clinicoradiographic comparison of the effects of platelet-rich fibrin gel and platelet-rich fibrin membrane as scaffolds in the apexification treatment of young permanent teeth.. J Indian Soc Pedod Prev Dent.

[19] Nadra M., Niu W., Kurisawa M., Rousson D., Spector M (2022). Platelet-rich plasma lysate-incorporating gelatin hydrogel as a scaffold for bone reconstruction.. Bioengineering (Basel).

[20] Tang S., Wang L., Zhang Y., Zhang F (2022). A biomimetic platelet-rich plasma-based interpenetrating network printable hydrogel for bone regeneration.. Front Bioeng Biotechnol.

[21] Sitkova E.S., Dragunova M.A., Ogurkova O.N., Smorgon A.V., Moskovskikh T.V., Batalov R.E., Suslova T.E (2023). Spontaneous and stimulated platelet aggregation activity in patients with atrial fibrillation and thrombotic complications.. Siberian Journal of Clinical and Experimental Medicine.

[22] Moskalensky A.E., Litvinenko A.L (2019). The platelet shape change: biophysical basis and physiological consequences.. Platelets.

[23] Murphy D.D., Reddy E.C., Moran N., O'Neill S (2014). Regulation of platelet activity in a changing redox environment.. Antioxid Redox Signal.

